# Evolution of Coagulation and Platelet Activation Markers After Transcatheter Edge-to-Edge Mitral Valve Repair

**DOI:** 10.3390/jcm14030831

**Published:** 2025-01-27

**Authors:** Sandra Hadjadj, Jonathan Beaudoin, Frédéric Beaupré, Caroline Gravel, Ons Marsit, Sylvain Pouliot, Benoit J. Arsenault, Philippe Pibarot, Julio Farjat-Pasos, Jorge Nuche-Berenguer, Benoît M-Labbé, Kim O’Connor, Mathieu Bernier, Erwan Salaun, Josep Rodés-Cabau, Jean-Michel Paradis

**Affiliations:** Quebec Heart and Lung Institute, Laval University, Quebec, QC G1V 4G5, Canada; sahad33@ulaval.ca (S.H.); philippe.pibarot@med.ulaval.ca (P.P.); benoit.m-labbe.med@ssss.gouv.qc.ca (B.M.-L.);

**Keywords:** transcatheter mitral valve repair, coagulation, platelets, antithrombin III, P-selectin

## Abstract

**Background/Objectives:** The recommendations for antithrombotic therapy after transcatheter edge-to-edge mitral valve repair (TEER) are empirical, and the benefit of antiplatelet (APT) or anticoagulation therapy (ACT) remains undetermined. The study sought to investigate the degree and the timing of coagulation and platelet marker activation after TEER. **Methods:** This was a prospective study including 46 patients undergoing TEER. The markers of coagulation activation, namely prothrombin fragment 1 + 2 (F1 + 2) and thrombin-antithrombin III (TAT), and the markers of platelet activation, namely soluble P-Selectin and soluble CD-40 ligand (sCD40L), were measured at baseline, 24 h, 1 month, and 1 year after TEER. **Results:** At discharge, 20 (43%) patients received APT (single: 16, dual: 4), 24 (52%) received ACT, and 2 (4%) had both single APT and ACT. Levels of F1 + 2 and TAT significantly increased at 24 h post TEER (both *p* < 0.001), rapidly returning to baseline levels at 1 month. However, levels of F1 + 2 and TAT remained higher at 1 month in patients without ACT compared to patients with ACT (respectively, 303.1 vs. 148.1 pmol/L; *p* < 0.001 and 4.6 vs. 3.0 µg/L; *p* = 0.020), with a similar trend at 1 year. Levels of soluble P-selectin and sCD40L remained stable at all times after TEER (respectively, *p* = 0.071 and *p* = 0.056), regardless of the APT. **Conclusions:** TEER is associated with an acute activation of the coagulation system, with no increase in platelet activation markers. Hence, the use of dual APT is questionable in this population. Our results raise the hypothesis that the optimal antithrombotic therapy after TEER could be short-term ACT over APT. Further larger studies are warranted.

## 1. Introduction

Transcatheter edge-to-edge mitral valve repair (TEER) is now widely established as a therapeutic option for high- or prohibitive surgical risk patients with symptomatic mitral regurgitation (MR) [1,2]. Although its safety and feasibility have been validated for both primary and secondary MR, complications increase morbidity and mortality [3,4]. Controlling the risk of cerebral vascular accident, device thrombosis, or systemic embolism versus bleeding episodes is fundamental to achieving successful procedural and clinical outcomes.

The current ACC/AHA and ESC guidelines do not provide recommendations for the management of antithrombotic therapy after TEER [1,2]. Actual treatment remains empirical and has been extrapolated from those previously used for similar procedures. One of the most commonly adopted treatment regimen is derived from the EVEREST (Endovascular Valve Edge-to-Edge REpair Study) study protocol and consists of a dual antiplatelet therapy (APT) composed of aspirin for 6 months to 1 year with clopidogrel for 1 month [4]. More recently, the COAPT (Cardiovascular Outcomes Assessment of the MitraClip Percutaneous Therapy for Heart Failure Patients With Functional Mitral Regurgitation) study protocol indicated the need for a single or dual APT if chronic oral anticoagulation therapy (ACT) was not used [5]. However, these regimens have never been evaluated in a randomized clinical trial. Therefore, the real benefit of using a single or dual APT over an ACT remains to be assessed.

So far, data regarding the thrombotic risk associated with the increase in the coagulation or the platelet biomarkers after a TEER are lacking, and there is no biologic basis supporting the actual antithrombotic regimens. Several biological markers are used to detect hemostatic changes. Prothrombin fragment 1 + 2 (F1 + 2) and thrombin-antithrombin III (TAT) reflect direct thrombin formation and thus coagulation activation, and were associated with hypercoagulability [6,7]. Soluble P-selectin (sP-selectin) and soluble CD40 ligand (sCD40L) have been well validated as markers of platelet activation, and were both found to be increased in diverse thrombotic scenarios [8,9,10,11]. Hence, the aim of this study was to prospectively investigate the degree and the timing of activation of these coagulation and platelet markers after the procedure.

## 2. Material and Methods

Study Design: Consecutive patients with moderate-to-severe and severe symptomatic MR who underwent TEER from May 2019 to November 2021 at the Quebec Heart and Lung Institute (Quebec, QC, Canada) were prospectively included in the present study. Either the MitraClip device (Abbott, Santa Clara, CA, USA) or the Pascal device (Edwards Lifesciences, Irvine, CA, USA) was implanted through transfemoral venous approach, under general anesthesia with fluoroscopic and transesophageal guidance [3,4,12]. Patients who had their intervention aborted, exhibited hemodynamic instability, or evidence of active infection at the time of treatment were excluded. Patients with no post-procedural complications were discharged 24 h after the procedure, and clinical follow-ups were scheduled at 1 month and 1 year after TEER. Blood samples were collected at baseline (the day or the morning before the procedure), 24 h after the procedure (before discharge) and during follow-ups at 1 month and 1 year. Primary endpoints were a change in prothrombotic status during the first year after TEER as determined by F1 + 2, TAT, soluble sP-selectin, and sCD40L. The secondary endpoints were defined according to the Mitral Valve Academic Research Consortium (MVARC) criteria [13]. All subjects involved in this study gave written, informed consent.

Antithrombotic Agents Administration: Patients receiving warfarin or a non-vitamin K antagonist oral anticoagulant were asked to stop their ACT 3 and 2 days prior the procedure, respectively. TEER was performed under anticoagulation with intravenous unfractionated heparin (100 U/kg), targeting an activated coagulation time above 250 s. The effect of heparin was neutralized and reversed using 50 mg of protamine sulfate at the end of the procedure. The antithrombotic therapy after TEER was left at the physician’s discretion. When dual APT was selected, aspirin 80 mg/day + clopidogrel 75 mg/day were prescribed for 30 days, followed by lifelong aspirin. For patients with high bleeding risk (HAS-BLED score > 3), single APT was an alternative option. For patients already on ACT prior to TEER, the same regimen was started the day after the procedure, if no bleeding or vascular complication had occurred. Patients received their medication the next morning after the procedure, before the 24 h blood collection and, if applicable, dual APT was stopped after the 1-month blood collection.

Assessment of coagulation and platelet activation: Blood samples were collected at specific times into 4 Vacutainer tubes prefilled with 0.5 mL of 3.2% buffered sodium citrate (Becton Dickinson) and immediately centrifuged at 1107× *g* at 4 °C for 10 min. Plasma was stored in plastic vials in aliquots at −80 °C until analysis. Enzyme immunoassays were used for determining concentration levels of (1) TAT (Siemens Healthcare Diagnostics Products GmbH, Marburg, Germany), (2) F1 + 2 (Siemens Healthcare Diagnostics Products GmbH, Marburg, Germany), (3) sP-selectin (R&D Systems, Minneapolis, MN, USA), and (4) sCD40L (R&D Systems, Minneapolis, MN, USA).

Statistical analysis: Continuous variables are presented as mean ± standard deviations (SDs). Categorical variables are reported as numbers and percentages. A mixed effect analysis using ANOVA for repeated measures was used to compare biomarker concentrations at different times. Posteriori comparisons were performed using Tukey’s method. The normality assumption was validated using the Shapiro–Wilk test on residuals from the statistical model. Either the one-way independent Student’s *t*-test or Mann–Whitney test was used to compare the differences in continuous variables between two groups of patients. The degree of activation of markers was expressed as percentage changes derived from the following formula = [(value at 24 h—value at baseline)/value at baseline] × 100. A *p*-value of <0.05 was considered significant. All analyses were performed using GraphPad version 9.4.1 (GraphPad Software, San Diego, CA, USA).

## 3. Results

Patient characteristics: During the study period, a total of 46 patients (14 women [30%]; mean age, 77 ± 8 years) were included in the study. The clinical and procedural characteristics of the population are displayed in Table 1. A high proportion of patients had atrial fibrillation (AF) at baseline (26 [57%]). The mean CHA_2_DS_2_-VASc score of those patients was 5 ± 1. A previous myocardial infarction was reported in 15 patients (33%), while 10 patients (22%) had at least one episode of stroke or transient ischemic attack. MR etiology was organic in 28 patients (61%), functional in 15 patients (33%), and mixed in 3 patients (6%). During the procedure, five patients (11%) had 3 clips implanted, whereas the average was 2 ± 1 clips. Patients were discharged after a median of 2 (1–2) days of hospitalization, most of them being admitted the day before the intervention. A third of them (14 [30%]) were treated with single APT before the procedure. At discharge, 16 patients (35%) were on single APT (aspirin in 15 patients [33%] and clopidogrel in 1 patient [2%]), and 4 patients (9%) were on dual APT. The majority of our cohort (>50%) was treated with ACT before and after the procedure. At discharge, two patients (4%) were treated with a clopidogrel + ACT (Table 2). Patients who received an ACT at discharge had more AF (*p* < 0.001) and significant higher levels of NT-proBNP (*p* = 0.032) at baseline than patients who received an APT (Table 1). There was no other difference in terms of comorbidities, cardiac function, MR etiology, or procedural characteristics between the two groups.

**Figure 1:**
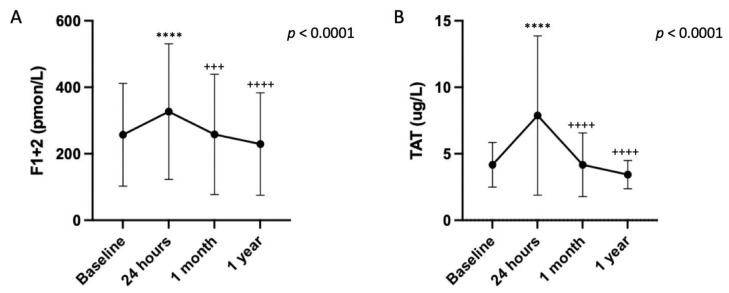
Changes in the markers of the coagulation activation after TEER. Changes in (**A**) pro-thrombin fragment 1 + 2 (F1 + 2) levels, and (**B**) thrombin-antithrombin III (TAT) levels showing a significant increase at 24 h after TEER to return to baseline levels at 1 month and 1 year in both cases. Data are expressed as mean ± SD. ^****^ *p* < 0.0001 vs. baseline; ^+++^ *p* < 0.001, ^++++^ *p* < 0.0001 vs. 24 h.

Coagulation activation biomarkers variation after TEER: The results of the coagulation system activation as assessed by F1 + 2 and TAT markers are represented in Figure 1. In our cohort of 46 patients, baseline levels of F1 + 2 and TAT were 230.7 (117.0–349.3) pmol/L and 3.5 (2.9–5.1) µg/L, respectively. There was an acute and significant change in the level of F1 + 2 and TAT at 24 h after TEER. Their levels increased up to 285.3 (186.4–409.0) pmol/L and 5.6 (3.9–9.8) µg/L (both *p* < 0.001), respectively. At that time, the increase from baseline values, as estimated from percentage changes calculated from repeated measurements analysis, was 33% (95% confidence interval [CI] 18 to 49) for F1 + 2 and 87% (95% CI 48 to 125) for TAT. In both cases, levels rapidly returned to baseline levels at 1 month, to remain stable at 1 year.

**Figure 2:**
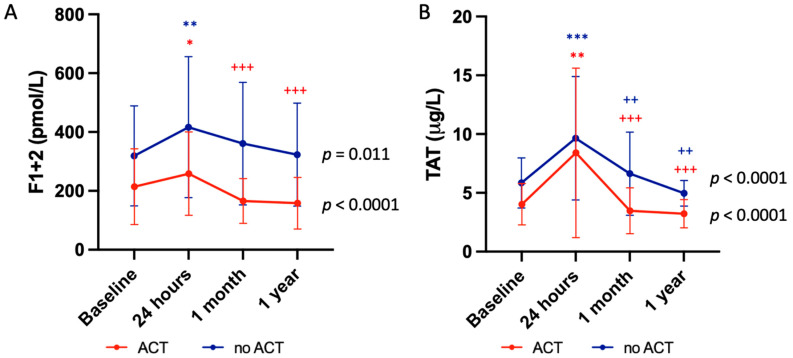
Changes in the markers of the coagulation system in regard to the antithrombotic treatment of the patients after TEER. (**A**) Changes in prothrombin fragment 1 + 2 (F1 + 2) levels showing a significant increase at 24 h after TEER followed by a significant decrease at 1 month and 1 year only in patients who were prescribed an anticoagulation therapy (ACT) in red compared to patients who were not prescribed any (no ACT) in blue. (**B**) Changes in thrombin-antithrombin III (TAT) levels in patients showing a significant increase at 24 h after TEER followed by a significant decrease at 1 month and 1 year whether patients were prescribed an ACT in red or not in blue. Data are expressed as mean ± SD. * *p* < 0.05, ** *p* < 0.01, *** *p* < 0.001 vs. baseline; ^++^ *p* < 0.01, ^+++^ *p* < 0.001 vs. 24 h.

Figure 2 illustrates the variation in marker levels depending on the anticoagulant regimen that was given to the patients after the procedure (ACT versus no ACT). There was a significant increase in the concentration of F1 + 2 at 24 h after TEER in both groups. Yet, the decrease at 1 month was only significant in patients who were prescribed an ACT at discharge (208.0 [158.3–319.7] vs. 148.1 [108.8–216.3], *p* = 0.001) compared to patients who were not prescribed any ACT (345.0 [250.0–482.9] vs. 303.1 [202.1–484.9], *p* = 0.431). Results were similar at 1 year. Patients with no ACT had overly higher levels of F1 + 2 than patients with ACT at 1 month (303.1 [202.1–484.9] vs. 148.1 [108.0–216.3], *p* < 0.0001) and 1 year (284.9 [201.3–405.9] vs. 130.7 [107.2–174.7], *p* < 0.001; Table 3). TAT levels were also significantly increased at 24 h after the procedure in both groups (Figure 2). The concentration drop was significant in both groups whether patients had an ACT (5.5 [3.6–10.6] vs. 3.0 [2.5–3.7], *p* = 0.018) or not (5.7 [4.3–9.5] vs. 4.6 [2.8–5.4], *p* = 0.011). However, TAT levels remained higher at 1 month in patients without ACT compared to patients with ACT (4.6 [2.8–5.4] vs. 3.0 [2.5–3.7]; *p* = 0.020), with a similar trend at 1 year (*p* = 0.136; Table 3).

The degree of activation of the coagulation markers according to baseline and procedural variables is displayed in Table S1. The increase in TAT levels between 24 h post-TEER and baseline tends to be larger in patients older than 78 years (median age of our cohort), though it did not reach a significant difference (≥78 years: 123 ± 159% vs. <78 years: 54 ± 65%, *p* = 0.068). Neither clinical nor procedural results variables correlated with the degree of activation of F1 + 2 and TAT levels in our cohort.

**Figure 3:**
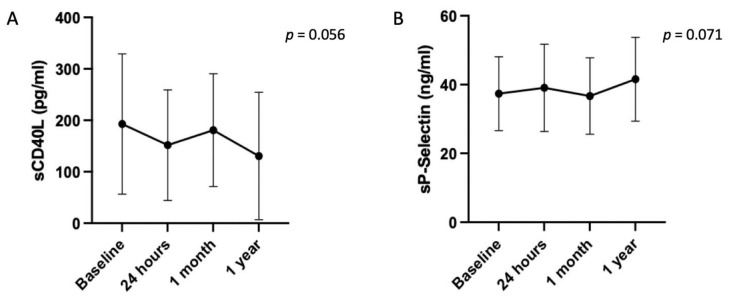
Changes in the markers of the platelet activation after TEER. Changes in (**A**) soluble CD40 ligand (sCD40L) levels, and (**B**) soluble P-selectin (sP-selectin) levels after TEER showing no significant variation in both cases. Data are expressed as mean ± SD.

Platelet activation biomarkers variation after TEER: The results of the platelet activation system as assessed by sCD40L and sP-selectin markers are represented in Figure 3. Baseline levels of sCD40L and sP-selectin were 154.3 (105.4–209.0) pg/mL and 35.1 (31.1–43.0) ng/mL, respectively. There were no statistically significant changes in the level of either marker at any time after TEER (sCD40L: *p* = 0.056; sP-selectin: *p* = 0.071). Using Turkey’s multiple comparisons test, both trends were defined by a decrease in sCD40L at 24 h, followed by a return to basal levels at 1 month (baseline vs. 24 h: 154.3 [105.4–209.0] vs. 142.7 [85.2190.0] pg/mL, *p* = 0.049; 24 h vs. 1 month: 142.7 [85.2190.0] vs. 161.7 [95.3–230.4] pg/mL, *p* = 0.033), and by an increase in sP-selectin levels at 1 year compared to baseline and 1 month (baseline vs. 1 year: 35.1 [31.1–43.0] vs. 42.1 [34.3–49.7] ng/mL, *p* = 0.008; 1 month vs. 1 year: 35.3 [29.7–42.1] vs. 42.1 [34.3–49.7] ng/mL, *p* < 0.0001).

**Figure 4:**
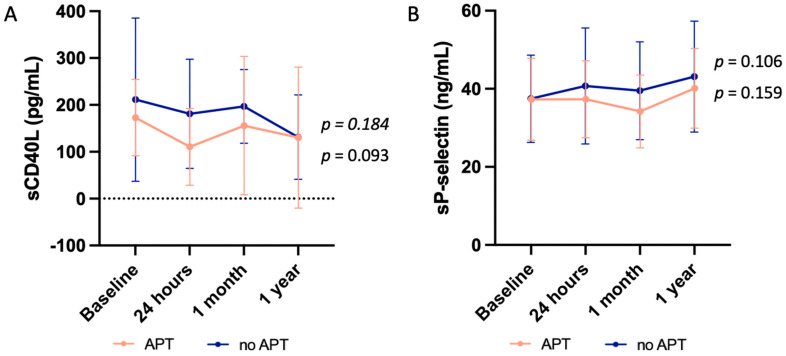
Changes in the markers of platelet activation in regard to the antithrombotic treatment of patients after TEER. Changes in (**A**) soluble CD40 ligand (sCD40L) levels, and (**B**) soluble P-selectin (sP-selectin) levels in patients who were prescribed an antiplatelet therapy (APT) in orange or not (no APT) in blue showing no significant variation in all cases. Data are expressed as mean ± SD.

The results of platelet activation markers variation according to the prescription of APT or not at discharge are illustrated in Figure 4. No significant change in platelet activation was seen regarding the prescription of APT after TEER. The levels of both markers at a specific time after TEER did not differ with regard to whether patients received an APT or not after the procedure.

Clinical outcomes during follow-up: Clinical events during the follow-up period are listed in Table 4. After a mean follow-up of 352 ± 49 days, there was no episode of device embolization, or major bleeding. The next day, two patients (4%) had minor bleedings at the femoral venous access site and one patient (2%) had a right sylvian ischemic stroke without sign of hemorrhage. A right sylvian ischemic stroke was clinically diagnosed by a neurologist the day after the procedure and confirmed on a cerebral computed tomography. The patient had permanent AF at baseline and no thrombus had been seen during the procedural transesophageal echocardiography. The patient stayed hospitalized for 13 days. This patient demonstrated an increase in F1 + 2 and TAT levels from 230.0 to 304.4 pmol/L (32% increase) and from 3.0 to 5.7 μg/L (88% increase), respectively, at 24 h after TEER. Although F1 + 2 levels decreased at one month, TAT levels kept increasing up to 6.6 μg/L (116% increase) and the patient died 6 months after the procedure due to decompensated heart failure. In total, there were two (4%) cardiac deaths during follow-up and nine (20%) hospitalizations for heart failure.

## 4. Discussion

In this study, we prospectively investigated the degree and the timing of coagulation and platelet activation markers in patients who had a TEER. The major findings of this study are as follows: (1) coagulation biomarkers levels (F1 + 2 and TAT) were significantly increased 24 h after the procedure and returned to baseline levels at 1 month, which demonstrated an acute activation of the coagulation system; (2) patients who did not receive any ACT after the procedure remained with higher concentrations of those biomarkers during follow-up; and (3) TEER was not associated with increased levels of platelet biomarkers (sP-selectin and sCD40L), demonstrating the absence of significant platelet activation following the procedure.

Coagulation system activation: In our cohort, the increase in coagulation markers was not driven by the presence of residual MR or post-procedural mitral stenosis, implying the impact of other parameters in the process. Virchow’s triad consists of three main factors that contribute to thrombosis: (1) endothelial dysfunction or injury; (2) hypercoagulability; and (3) hemodynamic changes (stasis or turbulence). It is undeniable that the procedure itself induces endothelial damage through the manipulation of intracardiac catheters and the necessity of a transeptal puncture. In the context of transcatheter closure of atrial septal defect (ASD), it was shown that the presence of a residual shunt was associated with a higher increase in F1 + 2 levels [14]. Hence, although the transcatheter iatrogenic ASD is expected to recover naturally throughout the first month after TEER, its presence might induce flow turbulence that contributes to the acute activation of the coagulation system observed in our study. The implantation of devices plays an essential role as well in the hypercoagulability state after TEER. Interestingly, the clip arms and grippers are covered with polyester fabric to promote tissue in-growth. Studies have demonstrated that the device is completely covered with tissue at 4 weeks, and the thickening process continues up to 12 weeks [15]. Thus, the metal surface of the device is exposed to circulating blood during the first weeks after TEER and is much more thrombogenic during this period, which biologically correlates with enhanced thrombin generation [16]. In our study, half of the population already had AF, which is associated with higher risk for thrombus formation due to blood stasis and specifically, increased thrombin activity [17]. However, no exhaustive data regarding new onset arrythmias after the procedure were collected. Some patients might have experienced new-onset AF after TEER that was not clinically diagnosed and that could partially explain the increase in the coagulation system biomarkers. Moreover, multiple studies have demonstrated that patients with AF and trace to mild MR showed significantly higher concentrations of coagulation markers with increased incidence of thrombus formation in the left atrium compared to patients with moderate to severe MR [18]. It was then hypothesized that severe MR might be protective against thrombus formation in the left atrium through a high-flow wash-out jet and thrombogenesis inhibition. Consequently, by reducing MR severity during TEER, the protection against blood stasis could be lost, therefore increasing the potential risk of thrombus formation in patients with new onset AF. The results of our study are comparable to those obtained after similar procedures such as transcatheter patent foramen ovale (PFO), ASD, or left atrial appendage (LAA) closure [14,19,20]. Those studies also showed activation of the coagulation system within days following the procedures, which completely returned to baseline values at 1 to 3 months post intervention. Similarly, after those procedures, there was no platelet activation either. Asmarats and colleagues have also shown that device-related thrombosis after LAA closure happened only in patients who were receiving APT. Those patients exhibited a greater coagulation activation at 7 days post LAAC [21]. In our cohort, correlation between coagulation markers and thrombotic events could not be assessed. However, we can hypothesize that the risk of thrombotic events might be greater in patients with no ACT, who exhibited more elevated levels of F1 + 2 and TAT during follow-up.

Absence of platelet system activation: In our study, platelet activation markers levels were compared among patients who received or did not receive an APT at discharge, and levels remained stable in both cases. The lack of platelet activation was likely related to the timing of blood sample collection. P-selectin and CD40L levels would be expected to increase during the procedure following the access to the femoral vein and the transseptal puncture. However, the current data shows that levels are similar to baseline the next day. In the context of prothrombotic disorders, sP-selectin and sCD40L levels are known to be increased. Some studies looking at patients with atherosclerotic vascular disease or coronary artery disease have even shown increased platelet activation despite concomitant APT with aspirin. This finding might explain why dual APT with clopidogrel was initially advocated after TEER in EVEREST trials [22,23]. However, regarding the results of our study, the lack of platelet activation following TEER may question the need for a dual APT in patients undergoing TEER. Furthermore, clopidogrel seems to be effective to prevent platelet activation in coronary artery disease, yet it does not seem as effective after TEER, and after transcatheter PFO, ASD or LAA closure [24,25,26]. On the other hand, the significant activation of the coagulation system during the first weeks might support the use of a short-term ACT after TEER in patients without contraindications to anticoagulants.

Towards the use of an anticoagulant after TEER: ACC/AHA and ESC surgical guidelines already show a class IIa indication for short-term ACT using an antivitamin K (AVK) (INR 2.0 to 3.0) within the first 3 months after mitral valve repair or bioprosthetic valve replacement, even in patients with no additional thrombotic risk factors [2,27]. A comparative study demonstrated that the incidence of stroke within 30 days after TEER was significantly reduced in patients who were prescribed an AVK for 30 days, with no increase in bleeding complication as compared to comparative cohorts who received usual dual APT [28]. More recently, 646 consecutive patients treated with an ACT prior to TEER were enrolled in a study to compare clinical outcomes according to their antithrombotic treatment after the procedure: ACT alone, ACT + single APT, or ACT + dual APT. The results demonstrated a significant decreased risk for all-cause mortality in the group of ACT monotherapy [29]. The mechanistic observations highlighted by our study could have a substantial clinical impact considering there are clinical studies that already provide some evidence for better long-term outcomes in patients treated with an oral anticoagulant monotherapy after TEER. Nowadays, the real-world antithrombotic treatment prescribed after TEER is heterogenous, with a 41% rate of treatments that do not follow current practice standards [30]. Randomized clinical trials are needed to establish the right recommendations on antithrombotic treatment after TEER. Data are required to determine the real benefit of a dual APT over a single APT, and to determine the benefit of a short-term ACT, in a patient with no initial indication for it, over an APT.

## 5. Limitations

This prospective study included a limited cohort of patients. Although the sample size was adequate for an overall evaluation of the evolution of hemostatic markers after TEER, subgroups analysis should be interpreted with caution, requiring confirmation in larger studies. Statistical analysis studying the impact of a dual APT over a single APT on platelet biomarkers level could not be achieved due to the small number of patients under dual APT in our cohort. The impact of ACT or APT on clinical outcomes could not be assessed due to the small number of events. Exhaustive data regarding new onset of arrythmias are missing. In fact, there were no clinically relevant new arrythmias, and no Holter monitoring was performed to detect any silent paroxysmal arrythmias that could have influenced the marker levels. There are some heterogenous concentrations of markers for a specific time that could be due to either an inadequate extraction or manipulation of blood samples, or the time of drug intake, especially at 24 h after TEER. Cardiac catheterization might induce an activation of the coagulation system acutely after the procedure, but its impact should be temporary. Thus, having supplemental time points at 7 days and 3 months would have been interesting to validate the influence of device endothelization timing on coagulation activation.

## 6. Conclusions

This study demonstrated that enhanced thrombin generation is the main hemostatic effect associated with TEER. This was illustrated by a rapid and significant activation of the coagulation system, with no increase in platelet activation markers. Hence, the use of dual APT after TEER is questionable, reinterring the urgent need for randomized clinical trials to clarify whether short-term ACT or single APT is the most appropriate regimen in this population.

## 7. Clinical Perspectives

The antithrombotic therapy after TEER remains empirical. Biological data regarding hemostasis system activation following TEER is lacking. Our findings showed a significant activation of the coagulation system biomarkers after TEER, that remained elevated during the follow-up in patients who were not prescribed any ACT. These biological data question the actual necessity of a dual APT after TEER and emphasize the need for adequate powered randomized clinical trials to better assess the real benefits of APT over ACT after TEER.

## Figures and Tables

**Table 1:** Baseline and procedural characteristics of the study population based on the antithrombotic regimen at discharge.

Variables	All Patients *n* = 44 *	Patients with APT *n* = 20 (43%)	Patients with ACT *n* = 24 (52%)	*p* Value
Baseline Characteristics				
Age, years	77 ± 8	76 ± 8	77 ± 8	0.579
Sex, female	14 (30)	8 (40)	6 (25)	0.342
BSA, m^2^	1.9 ± 0.3	1.8 ± 0.2	1.9 ± 0.3	0.125
Hypertension	33 (72)	12 (60)	19 (79)	0.199
Atrial fibrillation	26 (57)	2 (10)	21 (91)	*<0.001*
Paroxysmal	11 (24)			
Permanent/Persistent	15 (33)			
Dyslipidemia	29 (63)	9 (45)	18 (75)	0.063
Diabetes	8 (17)	3 (15)	5 (21)	0.710
CRF	28 (61)	12 (60)	15 (63)	1.000
Coronary artery disease	19 (41)	10 (50)	7 (29)	0.218
Myocardial infarction	15 (33)	7 (35)	7 (29)	0.752
PAD	9 (20)	5 (25)	4 (17)	0.710
Stroke/TIA	10 (22)	4 (20)	6 (25)	0.734
Imaging				
LVEF, %	46 ± 13	48 ± 13	47 ± 14	0.787
LVEDV, mL	118 (85–168)	116 (74–169)	101 (85–156)	0.852
LAVi, mL/m^2^	49 ± 20	45 ± 23	52 ± 18	0.316
Laboratory tests				
eGFR, mL/min/1.73m^2^	57 (22–71)	59 (44–73)	56 (42–74)	0.946
NT-proBNP, pg/mL	2004 (1001–3699)	1145 (613–2167)	2110 (1734–3804)	*0.032*
Hb, g/L	127 (114–147)	126 (119–147)	138 (129–146)	0.898
Procedural characteristics				
Procedure duration, min	114 ± 60	99 ± 10	116 ± 46	0.239
Number of clip(s)	2 ± 1	2 ± 1	2 ± 1	0.807
Residual MR > moderate	8 (17)	3 (15)	5 (21)	0.710

ACT, anticoagulation therapy; APT, antiplatelet therapy; BSA, body surface area; CRF, chronic renal failure defined by eGFR < 60 mL/min/1.73m^2^; eGFR, estimated glomerular filtration rate; Hb, hemoglobin; IQR, interquartile range; LAVi, indexed left atrial volume; LVEDV, left ventricular end-diastolic volume; LVEF, left ventricular ejection fraction; MR, mitral regurgitation; NT-proBNP, natriuretic protein; PAD; peripheral artery disease PCI, prior percutaneous coronary intervention; SD, standard deviation; TIA, transient ischemic attack. * Two patients were under ACT + APT at discharge and were not included in the comparison. Variables are presented as number (%), mean ± SD, or median (IQR).

**Table 2:** Antithrombotic treatment at baseline and discharge.

Variables	All Patients (*n* = 46)	
	Baseline	Discharge
Antiplatelet therapy	14 (30)	20 (43)
Aspirin	12 (26)	15 (33)
Clopidogrel	1 (2)	1 (2)
Aspirin + Clopidogrel	1 (2)	4 (9)
Anticoagulation therapy	25 (54)	24 (52)
Warfarin	4 (9)	4 (9)
NOAC	21 (46)	20 (43)
Clopidogrel + Anticoagulant therapy	1 (2)	2 (4)
None	6 (13)	0

NOAC, non-vitamin K oral anticoagulant. Variables are presented as number (%).

**Table 3:** Comparison of F1 + 2 and TAT levels according to antithrombotic therapy.

		No ACT	ACT	*p* Value
F1 + 2 (pmol/L)	Baseline	327.7 (215.9–397.9)	202.8 (95.8–301.1)	0.003
	24 h	345.0 (250.0–482.9)	208.0 (158.3–319.7)	0.004
	1 month	303.1 (202.1–484.9)	148.1 (108.0–216.3)	<0.0001
	1 year	284.9 (201.3–405.9)	130.7 (107.2–174.7)	<0.001
TAT (μg/L)	Baseline	4.1 (3.0–5.9)	3.4 (2.8–3.4)	0.448
	24 h	5.7 (4.3–9.5)	5.5 (3.6–10.6)	0.966
	1 month	4.6 (2.8–5.4)	3.0 (2.5–3.7)	0.020
	1 year	3.9 (2.8–4.2)	2.9 (2.4–3.3)	0.136

ACT, anticoagulation therapy; F1 + 2, prothrombin fragment 1 + 2; IQR, interquartile range; TAT, thrombin-antithrombin III. *p* < 0.05 is considered to indicate statistical significance. Data are presented as median (IQR).

**Table 4:** Clinical outcomes of the study population.

Variables	Total Population (n = 46)	No ACT (n = 20)	ACT (n = 26)
Mean follow-up, days	352 ± 49		
Stroke/TIA	1 (2)	1 (5)	0
Device embolization	0	0	0
Systemic embolism	0	0	0
Bleeding events			
Minor	2 (4)	0	2 (8)
Major	0	0	0
Extensive	0	0	0
Life-threatening	0	0	0
Fatal	0	0	0
Death			
Cardiac death	2 (4)	2 (10)	0
Non-cardiac death	0	0	0
Hospitalization			
HF hospitalization	9 (20)	5 (25)	4 (15)
Non-HF hospitalization	5 (11)	2 (10)	3 (12)

ACT, anticoagulation therapy; HF, heart failure; TIA, transient ischemic stroke. Variables are presented as number (%).

## Data Availability

Data is unavailable due to privacy and ethical reasons.

## Supplementary Materials

The following supporting information can be downloaded at: https://www.mdpi.com/article/10.3390/jcm14030831/s1, Table S1: Degree of activation of the coagulation markers according to baseline and procedural variables.

## Abbreviations

ACT: Anticoagulation Therapy; APT: Antiplatelet Therapy; F1 + 2: Prothrombin Fragment 1 + 2; MR: Mitral Regurgitation; TAT: Thrombin-Antithrombin III; TEER: Transcatheter Edge-to-Edge Mitral Valve Repair; sCD40L: soluble CD40 Ligand; s-Pselectin: soluble P-selectin.
