# Supplementary material for: Evolution of Coagulation and Platelet Activation Markers After Transcatheter Edge-to-Edge Mitral Valve Repair
Source: J Clin Med. 2025 Jan 27;14(3):831. doi: 10.3390/jcm14030831 (PMC11818723; doi:10.3390/jcm14030831)
Supplement: Supplementary file 1 [file jcm-14-00831-s001.zip › jcm-3375718-supplementary.pdf]

Supplementary Tables

Supplementary Table S1: Degree of activation of the coagulation markers according to baseline and procedural variables

Variables	ΔF1+2 (%)	p value	ΔTAT (%)	p value
Age, years				
≥ 78 (n=20)	32 ± 55	0.891	123 ± 159	0.068
< 78 (n=23)	34 ± 46		54 ± 65	
Dyslipidemia				
Yes (n=27)	34 ± 42	0.985	65 ± 81	0.155
No (n=16)	33 ± 62		121 ± 168	
CRF				
Yes (n=27)	38 ± 52	0.478	92 ± 144	0.702
No (n=16)	26 ± 47		77 ± 79	
Atrial fibrillation				
Yes (n=25)	34 ± 55	0.984	99 ± 150	0.450
No (n=18)	33 ± 42		70 ±73	
CAD				
Yes (n=18)	39 ± 39	0.558	64 ± 64	0.311
No (n=25)	30 ± 56		103 ± 152	
LVEF, %				
< 50 (n=21)	37 ± 45	0.656	92 ± 122	0.790
≥ 50 (n=22)	30 ± 55		82 ± 126	
Stroke/TIA				
Yes (n=10)	12 ± 28	0.119	87 ± 180	0.983
No (n=33)	40 ± 53		86 ± 103	
APT at baseline				
Yes (n=14)	41 ± 29	0.464	56 ± 44	0.261
No (n=29)	29 ± 57		102 ± 145	
Procedural duration, min				
≥101(n=21)	30 ± 39	0.673	88 ± 100	0.956
< 101(n=22)	37 ± 59		86 ± 144	
Residual MR				
> moderate (n=7)	40 ± 46	0.713	115 ± 116	0.515
≤ moderate (n=36)	32 ± 51		81 ± 125	
Mitral stenosis				
TMG > 5mmHg (n=15)	28 ± 45	0.880	73 ± 90	0.760
TMG ≤ 5 mmHg (n=28)	30 ± 40		84 ± 126	

Legends as Tables 1 and 3. APT, antiplatelet therapy; TMG, mean transmitral gradient. $p<0.05$ is considered to indicate statistical significance.
